# Molecular cross-talk between long COVID-19 and Alzheimer’s disease

**DOI:** 10.1007/s11357-024-01096-1

**Published:** 2024-02-23

**Authors:** Magdalena Pszczołowska, Kamil Walczak, Weronika Misków, Katarzyna Antosz, Joanna Batko, Julia Karska, Jerzy Leszek

**Affiliations:** 1 Faculty of Medicine, Wrocław Medical University, Wrocław, Poland; 2 Clinic of Psychiatry, Department of Psychiatry, Medical Department, Wrocław Medical University, Wrocław, Poland

**Keywords:** Alzheimer’s disease, Dementia, Long COVID, SARS-CoV-2, FOCM, Microglia

## Abstract

The long COVID (coronavirus disease), a multisystemic condition following severe acute respiratory syndrome coronavirus-2 (SARS-CoV-2) infection, is one of the widespread problems. Some of its symptoms affect the nervous system and resemble symptoms of Alzheimer’s disease (AD)—a neurodegenerative condition caused by the accumulation of amyloid beta and hyperphosphorylation of tau proteins. Multiple studies have found dependence between these two conditions. Patients with Alzheimer’s disease have a greater risk of SARS-CoV-2 infection due to increased levels of angiotensin-converting enzyme 2 (ACE2), and the infection itself promotes amyloid beta generation which enhances the risk of AD. Also, the molecular pathways are alike—misregulations in folate-mediated one-carbon metabolism, a deficit of Cq10, and disease-associated microglia. Medical imaging in both of these diseases shows a decrease in the volume of gray matter, global brain size reduction, and hypometabolism in the parahippocampal gyrus, thalamus, and cingulate cortex. In some studies, a similar approach to applied medication can be seen, including the use of amino adamantanes and phenolic compounds of rosemary. The significance of these connections and their possible application in medical practice still needs further study but there is a possibility that they will help to better understand long COVID.

## Introduction

In the last couple of years, medicine around the world faced a huge challenge—dealing with the pandemic caused by severe acute respiratory syndrome coronavirus-2 (SARS-CoV-2). One of the most widespread problems is long coronavirus disease (COVID), which is a multisystemic condition following SARS-CoV-2 infection. It is defined by WHO as the continuation or development of new symptoms, occurring 3 months after the initial infection, lasting at least 2 months, and having no other explanation, while the Center for Disease Control and Prevention (CDC) defines it as signs, symptoms, and conditions that continue or develop after acute COVID-19 infection. There are several hypotheses about the mechanism of long COVID. They include immune dysregulation, microbiota dysbiosis, autoimmunity, blood clotting, endothelial abnormalities, dysfunctional neurological signaling, disruption of blood–brain barrier, and possible reactivation of latent herpesviruses. [1, 2] Long COVID symptoms affect numerous organs and systems such as the heart, blood vessels, lungs, kidneys, liver, gastrointestinal tract, and reproductive, immune, and neurological systems. [1] Our study focuses on disorders and manifestations within the nervous system such as insomnia, fatigue, cognitive impairment called “brain fog,” anosmia, memory loss, depression, and anxiety. [1, 2] Some of these symptoms might resemble those observed in patients with Alzheimer’s disease (AD).

AD is a condition in which neurons are damaged by the accumulation of amyloid beta and hyperphosphorylated tau proteins. The amyloid beta interferes with transmission between synapses, while hyperphosphorylated tau proteins block the transportation of nutrients and molecules nourishing neurons. The presence of these proteins may activate microglia cells which cause chronic inflammation. All these processes lead to a decrease in brain volume. AD is the main cause of dementia. Its symptoms among others are cognitive impairment, memory loss, decreased judgment, and changes in personality, mood, and behavior [3].

Some studies have found that there are similarities between long COVID and Alzheimer’s disease, such as the mechanism, molecular pathways, changes in medical imaging, or applied medication. Our study is intended to summarize and analyze the significance of these connections.

## COVID-19 and AD connections (main facts)

Severe acute respiratory syndrome coronavirus-2 (SARS-CoV-2) is a lethal virus that was detected back on 31st December 2019 in Wuhan, Hubei Province in China. The pandemic disease was declared by the World Health Organization on 11th March 2020. In the middle of the pandemic, patients with AD, once infected by coronavirus SARS-CoV-2, are five times more likely to die of this infectious disease. [1] It was postulated that APOE4 (italic for gene), one of many important risk factors for AD, [2] would be a biomarker for severe COVID-19 [3]. Especially the type 4 allele of the gene for apolipoprotein E (APOE ɛ4) is a major susceptibility factor for AD and COVID-19 [4]. There is a link between APOE-ε4 and the severity of COVID-19. APOE-ε4 homozygotes were more exposed to severe COVID-19 infection and are more likely to have positive COVID-19 tests in comparison to e3e3 homozygotes [3]. Research has proven that the APOE genotype determines pathogen susceptibility or pathogen disabling in many infectious diseases. Protein products of the ApoE cluster genes may act as SARS-CoV-2 receptors since they have been proven to be receptors for various viruses such as herpesvirus or hepatitis virus C [5]. Pre-existing dementia and delirium are risk factors for the severity of COVID-19. SARS-CoV-2, similar to Alzheimer’s disease, causes neurocognitive disorders, olfactory dysfunction, excessive fatigue, and anxiety symptoms, and autopsies of COVID-19 patients’ brains have shown various types of neuropathological damage. [6] COVID-19 brains have shown degeneration and extensive inflammation, including those of people without neurological symptoms, and an overlap was found between marker genes of AD and genes that are upregulated in COVID-19 infection [7]. Inflammatory biomarkers such as TNF, interleukin-6, interleukin-1, complement proteins, and galectin-3 have been proposed as common prognostic biomarkers between SARS-CoV-2 infection and AD [8]. Tau aggregation and neurodegeneration can be caused by the activation of the NLR family pyrin domain containing 3 (NLRP3 inflammasome), triggered during SARS = CoV-2 infection [9].

Another hypothesis suggests that the potential increase in AD risk in COVID-19 patients could be related to Aβ, which can act as an antimicrobial peptide. It could be postulated that the SARS-CoV-2 neuroinvasion could promote Aβ generation, as part of the immune response, and the amyloid beta cascade leading to extracellular amyloid beta deposition [10].

In comparison to control, AD patients represent increased levels of angiotensin-converting enzyme 2 (ACE2) receptor expression. [8]. The ACE2 receptor is not age-dependent which suggests that there is a connection between ACE2 expression and AD [11]. ACE2 is expressed on neurons and glial cells, among others, on the temporal lobe and hippocampus—regions that are involved in the pathogenesis of AD [8]. Recent studies revealed that the ACE2 receptor might be a major cell entry receptor for SARS-CoV-2 [12]. The expression of ACE2 in the epithelium and endothelium describes the pathways of SARS-CoV entry and allows us to understand the pathogenesis of the disease [13]. ACE2 is present in human arterial and venous endothelial cells, small intestinal enterocytes, and epithelial cells of the lung [14]. In the nervous system, there is low or medium ACE2 expression. Affecting smell sense during COVID-19 may suggest that the virus may enter the brain via the nasal cavity through the olfactory nerve. In neurodegenerative disorders, such as Alzheimer’s disease, the virus can infect the brain through a disrupted blood–brain barrier [15]. Based on immunofluorescence staining and single-cell gene atlas study, ACE2 was found to be expressed mainly in type II alveolar epithelial cells and apical airway epithelial cells in humans [14]. This proved that measuring ACE2 in a saliva sample can be a valuable indicator in the diagnosis of COVID-19 infection because ACE2 is abundantly expressed in the surface epithelial cells of the oral mucosa and exfoliated epithelial cells in saliva [16]. The imbalance between ACE and ACE2 hurts endothelial dysfunction in COVID-19 patients with diabetes and hypertension [17]. Elevated plasma angiotensin II levels have been observed in COVID-19 patients. The binding of the SARS-CoV-2 virus to ACE2 causes a decrease in ACE2 levels and an increase in the ACE/ACE2 ratio due to the assumption that there was competition between angiotensin II and SARS-CoV-2 for binding to ACE2. The occupation of ACE2 by the virus may result in a decrease in the conversion of angiotensin II to angiotensin 1–7, which has health-promoting effects, including vasoprotective and anti-inflammatory effects [18]. Consequently, ACE/ACE2 enhancement may increase inflammation due to increased production of ACE-dependent cytokines and oxidative stress, and reduced counteraction mediated by ACE2 products [19]. It means that increased ACE2 expression could be a risk factor for SARS-CoV-2 in AD patients [20].

## Molecular pathways

### Impaired folate-mediated one-carbon metabolism (FOCM)

It was proven that both in patients with Alzheimer’s disease and patients with long COVID there are some misregulations in folate-mediated one-carbon metabolism (FOCM) [21]. FOCM may be described as metabolic connections between many pathways in the cell, which take place in the different departments of it—cytoplasm, mitochondria, or nucleus. This system is responsible for the serine and glycine interconversion, conversion of homocysteine to methionine, and the synthesis de novo of purines and thymidylate (dTMP) [22]. This is a network of dependencies based on one-carbon units (1C), which are obtained from serine and glycine catabolism. These units are later carried and activated at the oxidation states. The very important in these transformations are folate cofactors whose number is limited in the cytoplasm. It leads to the rivalry among biosynthetic reactions dependent on folate cofactors. Cells may regulate the flow of the 1C as a response to the stress conditions [23, 24].

To obtain optimal parameters by the cell, it is crucial to regulate the folate cofactors among FOCM pathways. The adjustment of the level of transcription of the enzymes of the FOCM network allows control of metabolism. An increased homocysteine level may be an indicator of FOCM impairment [22].

Since SARS-CoV-2 affects almost all parts of the human body, including the brain, it was proven that it also affects the FOCM network. During intensive replication, the SARS-CoV-2 virus changes the cell’s metabolism to deliver methyl groups [25]. It results in a decreased amount of methyl groups in the cell but also causes oxidative stress which leads to reactive oxygen–nitrogen species (RONS). SARS-CoV-2 in the cell may also lead to a decrease in the level of homocysteine and glutathione. What is more, it may oxidize cofactor cobalamin (vitamin B_12_). Previously described changes lead to the impairment of the FOCM pathways which may evoke neuronal cell damage. This may lead to neuroinflammation and diminished cognition [26].

**Fig. 1:**
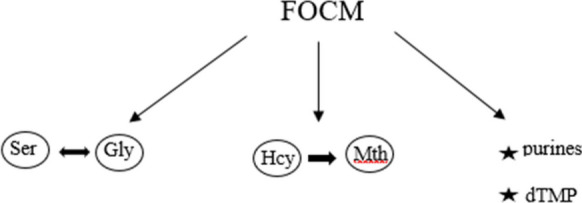
FOCM (folate-mediated one-carbon metabolism). Ser serine, Gly glycine, Hcy homocysteine, Mth methionine, dTMP deoxythymidine monophosphate

In Alzheimer’s disease, the level of the homocysteine in the cells is also elevated which may cause the impairment of the FOCM [27]. In these conditions, there are also RONS elevated due to the oxidation stress [22]. According to some research, it is possible to decrease the level of homocysteine due to regular oral supplementation of vitamins B_6_ and B_12_ and what is more the decrease of the rate of growth of amyloid-β1-40. [28] The above dependencies are presented in Fig. 1.

### Ubiquinone (Cq10)

An essential endogenous antioxidant in the human body is thought to be Cq10, also known as ubiquinone. Cq10 is responsible for reducing ROS and the regeneration of antioxidants such as vitamin E [29]. Ubiquinone appears in all organisms’ membranes and plasmas, with the highest concentration in mitochondrial membranes and highly metabolic organs in general. [30, 31] The level of Cq10 depends on various factors including age, hormones, diet, gender, or exercise [31–37]. The deficiency of Cq10 may be explained by a mutation in genes encoding Cq10 or elements of pathways in which disturbance results in OXHOS defects and lower levels of Cq10 consequently. Certain diseases or drugs such as statins could decrease the synthesis of Cq10 as well [37–39]. Referring to the literature, and previous studies that have examined the consequences of Cq10 deficiency, this state could result in a variety of disturbances in highly metabolic tissues including the brain, kidneys, and skeletal muscles. Cerebellar ataxia, encephalopathy, myopathy, or nephrotic syndrome are highlighted examples [29].

The deficit of Cq10 may occur in both AD and myalgic encephalomyelitis/chronic fatigue syndrome (ME/CFS) [40–43]. ME/CFS is an infection-linked disease and it presents a similar range of symptoms as long COVID—“brain fog,” dysrhythmias, pain, or breathlessness persisting months after the period of acute infection [43, 44]. This association between AD and the disease resembling long COVID may suggest possible interactions between these disorders. Taking into consideration recent studies, Cq10 could be a potent therapeutic agent against AD, ME/CFS, and/or long COVID [45].

### Microglia

Microglia cells play an important role in brain development, neuronal networks, and injury repair processes. A body of literature on long COVID shows the phenomenon of the white matter–selective microglial reactivity during long COVID after respiratory COVID-19. COVID-19 may result in a certain transcriptional state in microglia, and in the case of the severe course of the disease, decreased expression of homeostatic microglia genes [7, 46]. These changes present overlapping features with disease-associated microglia (DAM) typical for neurodegeneration in Alzheimer’s disease [47]. SARS-CoV-2 can directly infect human microglia, eliciting M1-like pro-inflammatory responses, followed by cytopathic effects [46].

A series of recent studies indicated that choroid plexus in individuals after severe respiratory COVID-19 may present an inflammatory state with specific chemokine signaling to cortical microglia, oligodendrocytes, astrocytes, and neurons, together with complement transmission of signals to cortical microglia [7]. The latter issue could resemble abnormal synaptic pruning by microglia triggered by complement signaling characteristics for Alzheimer’s disease [48]. Although studies have been conducted, this pathogenetic linkage between AD and long COVID seems to be still insufficiently explored and remains to be discovered.

## Endothelium impairment

The endothelium is the layer of the blood vessel that has the highest level of contact with blood is the epithelium that lines its inner site. Due to the constant contact of the endothelium with circulating blood, its cells are also connected in many processes that take part in this tissue, for example, inflammation process [49]. The etiological agent of COVID-19 is the SARS-CoV-2 virus which reacts with angiotensin-converting enzyme 2 receptor. This receptor occurs commonly in alveoli; however, it is also present in endothelial cells [50]. Increased level of pro-inflammatory factors damages the endothelium, as well as aging, increased levels of sex hormones, and ROS [51]. What is more, it is damaged by circulating endothelial microparticles (EMPs) and circulating progenitor cells (PCs) in the increased amount [52].

The consequences of SARS-CoV-2 infection on endothelial cells include hypoxia, inflammation, death of the cell, and renin–angiotensin system disorder [49]. Hypoxia is caused by increased ROS formation, higher levels of blood tackiness, and a decrease in intracellular pH. There are also signaling pathways activated. Factors like complement activation, RAS imbalance, or a high amount of cytokines are increasing the level of endothelial impairment [52]. Loss of normal functioning of the endothelium may lead to impaired vasodilation, extended pro-inflammatory and prothrombotic abilities, and irregular modulation of vascular growth [53]. Endothelium dysfunction is also characterized by enlarged levels of von Willebrand factor (VWF) and tissue factor (TF) which are pro-adhesive proteins. There are also more adhesion receptors at the cell surface [54]. Much research shows that endothelial damage is significant when it comes to the progression of COVID-19 [50, 55].

Protein-transmembrane protease serine 2 (TMPRSS2) which is present on endothelial cells allows the SARS-CoV-2 virus to enter the cell [56]. What is more, viral spike protein enables interaction with the endothelium cell through angiotensin-converting enzyme 2 (ACE2) which results in ACE2 downregulation. In effect, inflammatory pathways in the cell are activated [57]. Interestingly, during the SARS-CoV-2 inflammation, there is an enlarged risk of thrombosis occurrence and the levels of reactive C protein, ferritin, and D-dimer which are markers of acute state [50].

It was also discovered that COVID-19 leads to increased digestion of endothelial cell glycocalyx by heparanase 1 which simplifies virus entering the cell [58].

As COVID-19 is frequently characterized as an endothelial disorder, it is worth noting the intricacies of cerebrovascular endothelial dysfunction concomitant with cognitive impairment. Cerebrovascular endothelial dysfunction denotes an impairment in the regulatory functions of endothelial cells lining cerebral blood vessels. These cells are integral to vascular homeostasis, governing blood flow dynamics, permeability across the blood–brain barrier (BBB), and the synthesis of signaling molecules [59].

The intricate relationship between cerebrovascular endothelial dysfunction and cognitive impairment stems from several interconnected mechanisms.

Endothelial cells orchestrate hemodynamic regulation through nitric oxide–mediated vasodilation. Dysfunction in these cells compromises this regulatory mechanism, resulting in suboptimal cerebral blood flow, thereby diminishing the supply of oxygen and nutrients to the brain and potentially precipitating cognitive decline [60].

The endothelial cells constitute the BBB, crucial for shielding the brain from systemic insults [61]. Dysfunction in these cells compromises the BBB’s structural and functional integrity, facilitating the ingress of potentially neurotoxic substances [62]. This breach elicits inflammatory cascades, influencing cognitive function [63].

Endothelial dysfunction is conducive to heightened vascular inflammation. Prolonged inflammatory states are implicated in neurodegenerative pathologies such as Alzheimer’s disease and vascular dementia, contributing to cognitive compromise [64].

Impaired endothelial function augments oxidative stress, manifesting as an imbalance between reactive oxygen species and antioxidants. Excessive oxidative stress inflicts cellular damage, including neuronal injury, thereby contributing to cognitive deterioration [65].

Endothelial dysfunction is often associated with small vessel disease, characterized by cerebral microvascular abnormalities. This vascular pathology precipitates lacunar infarcts and white matter lesions, instigating cognitive decline [66].

Optimal endothelial function is imperative for neurovascular coupling, the synchronized adjustment of blood flow to neuronal activity. Dysfunction in this coordination may lead to inadequate perfusion during cognitive tasks, influencing cognitive performance adversely [67, 68].

Finally, endothelial dysfunction is considered a predisposing factor for neurodegenerative diseases, including Alzheimer’s disease. While the precise mechanistic interplay is incompletely elucidated, vascular elements are thought to synergize with other neuropathological processes [69].

There is emerging evidence supporting endothelial dysfunction in the context of long COVID, accompanied by cognitive impairment. In the long COVID, the essential immune and inflammatory response persists, leading to sustained systemic inflammation marked by elevated cytokines, including IFN-β, IFN-λ1, IFN-γ, IL-2, IL-6, IL-17, CXCL8, CXCL9, and CXCL10 [70]. This extended cytokine release activates specific immune cell populations, such as non-classical and intermediate monocytes, as well as other cell types like fibroblasts and myeloid cells. An aberrant Th2 cytokine pool induces CCL11 production, causing neuroinflammation with activated microglia releasing increased CCL11. This process contributes to reduced hippocampal neurogenesis, loss of myelinating oligodendrocytes and oligodendrocyte precursors, and subsequent subcortical white matter demyelination [71]. These mechanisms are strongly associated with cognitive impairments and neuropsychiatric symptoms [46].

In the context of long COVID and associated cognitive symptoms often referred to as “brain fog,” the presence of microscopic thrombi or “micro-clots” in the cerebral vasculature may explain cognitive impairments. These impairments are posited to result from the destruction of small-fiber nerve cells and the onset of dysautonomia [72].

Moreover, in human neural organoid models, it has been demonstrated that the occurrence of the neurological implications of long COVID may be associated with a substantial expression of ACE2 receptors on the apical aspect of the choroid epithelium [73]. This receptor distribution facilitates the invasion of SARS-CoV-2 through the vasculature, leading to subsequent ependymal cell death and disruption of the blood–cerebrospinal fluid barrier (B-CSF-B) [74].

Furthermore, after B cell activation in the peripheral system and cytokine dysregulation, the resultant serologic IgG and IgA antibodies display a polyclonal distribution, influencing cytokine function and endothelial integrity. Notably, these antibodies can permeate the central nervous system (CNS) due to blood–brain barrier (BBB) disruption [67]. While reports are limited, these autoimmune responses are suggested to accompany conditions including neuropsychiatric symptoms [75]. Prolonged antinuclear antibody (ANA) autoreactivity has been associated with enduring symptoms of dyspnea, fatigue, and cognitive impairment observed in individuals with long COVID [76].

A leading proposed mechanism underlying long COVID symptoms involves the activation of the neuroimmune system via the interaction between neural cells and glial cells, specifically astrocytes, microglia, and oligodendrocytes. Substantiated by heightened levels of ezrin (EZR) in individuals with long COVID, these activated astrocytes elevate NF-κB expression [77, 78]. This upregulation is implicated in endothelial cell death and augmentation of extracellular glutamate levels, leading to blood–brain barrier disruption and neurodegeneration induced by hyperexcitability, respectively [79].

Additionally, utilizing flow cytometry on peripheral blood from individuals with long COVID revealed heightened expansion of non-classical monocytes (CD14dimCD16 +) and intermediate monocytes (CD14 + CD16 +) up to 15 months post-infection compared to healthy controls [80]. Non-classical monocytes play a physiological role in complement-mediated and antibody-dependent cellular phagocytosis against viral insults, commonly residing along the luminal side of vascular endothelium, thereby contributing to blood–brain barrier (BBB) integrity. Notably, severe long COVID cases exhibited increased levels of macrophage scavenger receptor 1 (MSR1), indicating elevated peripheral macrophage activation, potentially disrupting the BBB and causing tissue damage [79]. Conversely, intermediate monocytes specialize in antigen presentation and simultaneous secretion of pro-inflammatory cytokines. While this systemic hyperinflammatory state has not been directly implicated in neuropsychiatric manifestations, it may contribute to disease progression through chronic activation of specific monocyte and T cell populations and neurovascular dysfunction of the BBB. These mechanisms can lead to the dissemination of inflammatory molecules and immune cells from the periphery into the central nervous system, inducing a persistent neuroinflammatory response [77, 78].

Collectively, endothelial-associated mechanisms can result in the dissemination of inflammatory cytokines and immune cells into the CNS, infected leukocyte extravasation across the BBB, and microhemorrhage. These processes ultimately contribute to the underlying neurological and cognitive symptoms observed in long COVID. Additional clinical, neuropathological, and experimental models are necessary to address numerous unanswered questions regarding the pathogenesis of long COVID. Similarly, the effectiveness of current and potential therapeutics targeting these hypothesized pathogenic mechanisms using anti-inflammatory, antiviral, and neuro-regenerative agents could potentially reverse neurological sequelae. However, well-designed clinical trials are still required to substantiate their efficacy.

## Vascular pathology in Alzheimer’s disease and long COVID

Many studies have found that vascular pathology and age-related changes in blood vessels play a significant role in AD pathogenesis and may not only co-occur with AD but also precede its onset. The main processes that lead to neurodegeneration are blood–brain barrier (BBB) dysfunction, hypoperfusion-hypoxia, and cerebral amyloid angiopathy (CAA) [81, 82]. BBB breakdown is caused by the degeneration of the endothelium and pericytes, by the reduction in the expression of occluding ZOI, or claudin 5, which are tight junction proteins, and by increased bulk-flowed transcytosis. It can lead to the accumulation of molecules in the brain such as immunoglobulins, albumin, thrombin, or plasmin. That results in brain edema, neurotoxicity, memory impairment, and further vascular and BBB damage. In AD, this can lead to impairment in Aβ clearance contributing to its accumulation [83]. Hypoperfusion, called oligemia, has a deep impact on synaptic plasticity whereas hypoxia affects ATPase activity leading to a lower ability of neurons to generate action potentials. These processes lead to edema, lesions, and accumulation of neurotoxins such as Aβ and hyperphosphorylated tau proteins, which are responsible for AD pathogenesis. Hypoxia also induces an increase in β-secretase expression and, through MAPK, promotes phosphorylation. Thus, hypoperfusion can induce or amplify changes that lead to the development of AD [81]. Cerebral amyloid angiopathy (CAA) is a disease involving the build-up of Aβ deposits in blood vessels in the central nervous system (CUN) [84]. Many publications show that CAA is associated with a higher risk of progressing cognitive decline and brain atrophy. Most patients with AD have some degree of CAA and the apolipoprotein E (ApoE) ε4 allele is a risk factor for both CAA and AD [85]. Intracranial bleedings caused by CAA contribute to the progression of dementia [81]. Long-term effects of COVID lead to endothelial dysfunction and the progression of atherosclerotic plaques consisting of inflammatory cells, protein platelets, and macrophages leading to a decrease in cerebral blood flow and further to hypoperfusion and hypoxia [86]. These lead to previously discussed changes also seen in AD.

Cerebral small vessel disease (CSVD) is said to be one of the most common causes of vascular dementia (VaD) [87]. This is a condition that affects cerebral small arteries and microvessels. It is a group of various diseases characterized by small subcortical infarcts, lacunes, enlarged perivascular spaces, white matter hyperintensities, cortical atrophy, and cerebral microbleeds 66. A systematic review written by Owens et al. proved the development of CSVD in patients with a COVID-19 infection history. The risk of developing CSVD increases in patients over 55 years old and in patients suffering from hypertension or diabetes type 2 [88]. CSVD is characterized by some specific findings in MRI described further in this review.

## Therapy options

Nowadays, an optimized treatment for long COVID is one of the major issues in medicine. Therapy of Alzheimer’s disease in this approach seems to be inseparable from long COVID itself. However, even with all of this focus around, there is presently no treatment for long COVID that could affect different symptoms simultaneously. There is an urgent need to create a personal care plan to manage the symptoms and improve quality of life [89, 90].

However, knowing the pathogenic of COVID-19 as well as long COVID, it seems reasonable to focus on pharmacological agents for vascular disease that could benefit in the treatment or the prevention of chronic vascular complications. Moreover, therapies applied in myalgic encephalomyelitis/chronic fatigue syndrome (ME/CFS) also are used as a form of treatment for long COVID [90, 91].

Considering Alzheimer’s disease and long COVID therapeutic options, medications such as β-blockers, low-dose naltrexone, and intravenous immunoglobulin were tested due to their potential role as pharmacological agents. Low dose such as 3–4.5 mg of naltrexone has been used already in many countries for several diseases (including ME/CFS) due to its immune modulatory effect. This treatment shows promising results in treating long COVID as well [91, 92].

As another option for the prevention of vascular diseases and therapy for abnormal clotting, anticoagulants are also used. The results seem to be promising [93–95]. In one study trial, patients with long COVID were treated with “triple” therapy including antiplatelet therapy (DAPT—clopidogrel 75 mg and aspirin 75 mg) once a day, and a direct oral anticoagulant (DOAC—apixaban 5 mg) twice a day. The study proved a noticeable reduction in micro-clotting and platelet hyperactivation in imaging tests, as well as reductions in individual symptoms in the majority of patients [94]. Additionally to this study, there are more and more promises about using anticoagulation drugs in long COVID therapy. Early prophylactic can contribute to the prevention of vascular endothelium damage, reduce thrombotic sequelae, and improve a patient’s quality of life [95].

Anticoagulation is a standard element of COVID-19 treatment in hospitals almost from the beginning. There are plenty of studies that confirm their significance and also there is a strongly made assumption that early anticoagulation therapy can be beneficial in high-risk patients [95]. This leads to the appliance for prevention from long COVID, as anticoagulants can reduce vascular disease progression by thrombotic sequelae inhibition. However, the application of anticoagulation is limited, as in advanced disease these medications are not able to compete sufficiently with prothrombotic agents [95]. In this approach, vascular long COVID is considered an individual type of COVID-19 complication that can lead to plenty of vascular diseases [86], e.g., as in this paper—Alzheimer’s disease.

Referring to the idea of reducing the level of antibodies as well as eliminating micro-clots, apheresis could be considered. However, due to being a rather expensive option, it is not broadly used [96].

Since long COVID is a worldwide complication, researchers are trying very different approaches to cure it. Therefore, there are plenty of ideas, such as using supplements (e.g., coenzyme Q_10_ and d-ribose) but most of these studies may require further data [45, 90].

Besides supplements, another common belief was already examined. The study focused on the relationship between physical activity and long COVID shows very mixed effects [97, 98]. Although some people may achieve improvement in their condition, even 75% of patients could worsen by implying physical activity in their therapy [97]. Therefore, it could be used as an additional form of treatment, and some people may achieve improvement in their condition and alleviate the disease progression leading to long COVID-19 [90, 98].

Recent studies and case reports have suggested another treatment option. The application of antiviral Paxlovid not only for acute COVID-19 but also for long COVID is considered as Paxlovid may decrease long COVID by 25% [90].

Older drugs with antiviral potential have been considered lately, giving promising studies. The amino adamantanes such as amantadine and memantine seem to be in the spotlight when it comes to long COVID. They both are well-known *N*-methyl-d-aspartate antagonists used in Parkinson’s and Alzheimer’s diseases and therefore in neurodegenerative diseases [99].

This could be a very promising field of study while taking into account both the biochemistry of long COVID and these neurodegenerative diseases—Parkinson’s and Alzheimer’s [100]. Furthermore, comparing major and minor symptoms of neurodegenerative diseases with those linked with long COVID indicates plenty of common anchor points as well. This leads to the conclusion that amino adamantanes could be an effective alternative to alleviate or even cure post-COVID neurodegenerative complications [99, 101]. Nevertheless, this theoretical approach needed to be confirmed in clinical research.

Last but not least, natural medicine draws attention due to the enormous potential hidden in herbal extracts. Quoting Hippocrates, there is already a cure for every disease in nature. Among the latest studies, the one based on Rosemary’s healing properties raises hope on this subject.

Rosemary (*Rosmarinus officinalis* [family Lamiaceae]) is applied in many different countries as a traditional remedy. The immense diversity of potentially active compounds can be divided into two main groups. One group includes aromatic compounds (“essential oils”). The other group features polyphenolic compounds such as carnosic acid (CA) and carnosol (CS) that are proven to present antioxidant actions on very different levels [102].

This biomolecular potential of CA and CS could be applied in the treatment of Alzheimer’s and Parkinson’s disease, as well as in COVID-19 and long COVID. These phenolic compounds perform antioxidant and anti-inflammatory functions that among others give neuroprotective effect. This promising potential can be explained by the activation of the KEAP1/NRF2 transcriptional pathway that attenuates the activation of NLRP3 (a part of the multimeric protein complexes—inflammasomes). The inhibition of the NLRP3 inflammasome by CA and CS leads to the limitation of pro-inflammatory cytokine production (“cytokine storm”). In this context, the significant role of the NLRP3 inflammasome in neurological diseases such as AD and the pathogenesis of neurological long COVID symptoms is worth emphasizing [102, 103].

Furthermore, these rosemary-active compounds pass through the blood–brain barrier and have good bioavailability while administrated orally. Also, this described manifestation of the neuroprotective effect is already thoroughly known; therefore, this therapy risk seems to be lower than completely unfamiliar ones [102].

In conclusion, many reports have already appeared, and many will. Directions of treatment searching are strongly diverse and the science assignment is to find the most corresponding to the patient’s needs.

Possible therapy options are presented in Table 1.

**Table 1:** The summary of possible treatments according to the teams led by Pitt [92], Davis [90], Tölle [104], Wright [97], Müler [43], and Satoh [102]

No	The possible therapy	Results
1	Low-dose naltrexone	The immune modulatory effect
2	Anticoagulants	Reducing the vascular disease progression
3	apheresis	Promising effects but rather expensive
4	Supplements (e.g., coenzyme Q_10_ and d-ribose)	Most of the studies may require further data
5	Physical activity	Very mixed effects and not recommended in the majority of patients
6	Antiviral Paxlovid	As long COVID treatment decreases disease progression
7	Amino adamantanes (amantadine and memantine)	Alleviation or even cure post-COVID neurodegenerative complications
8	Polyphenolic compounds from rosemary extract	Anti-inflammatory functions with neuroprotective effect

## Imaging

There were several chances in medical imaging. There are many studies showing changes in the EEG profile of patients with COVID-19. In EEG, a lower delta band was shown and it was closely related to the severity of cognitive disturbances at follow-up [105]. Individual alpha frequency (IAF) at EEG baseline was lower in COVID-19 survivors than in healthy subjects [10]. Abnormality which appeared in the biggest number of patients was diffuse slowing. Patients also presented focal slowing, lethargic posterior dominant rhythm, background attenuation, and the absence of posterior dominant rhythm [106]. It also showed that delta bend has a high regional current density in long COVID patients [107]. Some studies showed that the supposed passage of SARS-CoV-2 to the brain may be connected to characteristic abnormalities in the frontal region [108, 109].

In the studies conducted by Appelt et al. in Brazil, it was proven that even after a few months since acute COVID-19 infection, there are changes in electric brain activity, which decreased at rest in the Fz–F4 zones and during high cognitive demanding tasks in the F3–F7 areas. What is more, the complexity was reduced in F3–F7 at rest [110]. The association between EEG complexity and cognitive decline was investigated, and they explain the reduction in complexity by the reduced number of neurotransmitters [111, 112]. What is important is that the changes in the brain do not only refer to patients with a severe variant of the COVID-19 infection [113].

In MRI, there were microbleeds in hospitalized patient’s brains presenting long COVID symptoms [114]. Additionally, the volume of gray matter decreases greatly, especially in the orbitofrontal cortex and the parahippocampal area, and the global brain size reduction was observed [115]. One study also revealed white matter lesions, with a propensity for a biparietal distribution [116]. Another research has found features of changes in medial temporal lobes occurring after COVID-19, as well as altered thalamic connectivity [117]. However, it is not always possible to notice improper findings in MRI in patients with cognitive impairment after SARS-CoV-2 infection [116]. What is more, MRI abnormalities were present in patients regardless of the severity of their condition. The findings in MRI occurred both in patients admitted to ICU and those with mild COVID-19 manifestation [118].

Also, patients suffering from CSVD present some characteristic findings in MRI such as new subcortical infarcts, lacunes, dilated perivascular spaces, white matter hyperintensities (WMHs), and cerebral microbleeds (CMBs) [119]. WHMs visible on T2 MRI are mostly bilateral in older individuals. There is also vessel wall thickening observed. Other changes in vessels like an increase in tortuosity and a decrease in density are also present. There is demyelination, gliosis, fiber loss, and decreased number of oligodendrocytes observed [120]. There are various names used by radiologists to describe such pathologies—Wardlaw et al. estimated the frequency of used expressions. The most common were leukoaraiosis, white matter lesions (WML), and white matter hyperintensity (WMH) [119].

Cerebral microbleeds (CMBs) observed in patients with CSVD are visualized as small regions of the signal void with associated blooming on the T2 MRI. There have been also pathologies like tissue loss and gliosis observed [120]. Histopathological examination revealed damage to the walls and micro-hemorrhages in the parenchyma [121].

Biomarkers like neurofibrillary tangle or cortical Aβ were found to have a positive correlation between AD and CSVD [120]. A prospective cohort study revealed in older adults an association between the WMH volume score in T2 MRI and the cerebral Aβ burden [122]. This was the strongest correlation between the investigated biomarkers [120]. Another multicohort study proved that CMBs are found in CSF more often in patients with AD than in patients without this condition [123].

Long COVID patients presented hypometabolism in the parahippocampal gyrus, thalamus, and cingulate cortex [124] using the FDG PET method. [125] In some patients whose MRI did not reveal any anatomical changes, there was an 18FDG PET/CT scan applied. It showed in a patient with anosmia symptoms that hypoactivity of the left orbitofrontal cortex was made visible, which was linked with the hypothesis of a neuroinvasive mechanism for anosmia in COVID-19 patients [126].

## Long COVID–AD: epidemiology

The retrospective study conducted by Taquet [57] has shown that among 273,678 COVID-19 survivors, 57% of them had long COVID symptoms for 6 months after COVID-19 infection and 36.55% of them had long COVID symptoms between 3 and 6 months after COVID-19 infection.

It was demonstrated that the most common long COVID symptoms that occurred between the 1st and 180th day of COVID-19 infection were anxiety (22.82%), abnormal breathing (18.71%), abdominal symptoms (15.58%), depression (15.49%), fatigue (12.82%), chest pain (12.60%), headache (8.67%), cognitive syndromes (7.88%), and myalgia (3.24%) [127].

The risk of the most common mental disorders—mood disorders and anxiety—caused by COVID-19 decreases significantly after 1–2 months and occurs in the same incidence as the negative control group after 417–457 days. However, the risk of cognitive and psychotic disorders and seizures is constantly increased after an acute period of COVID-19.

In the children group, it was noticed that there is an increased risk of cognitive disorder, insomnia, intracranial hemorrhage, psychotic disorder, and epilepsy but the same risk of anxiety and depression 6 months after COVID-19 [128].

**Fig. 2:**
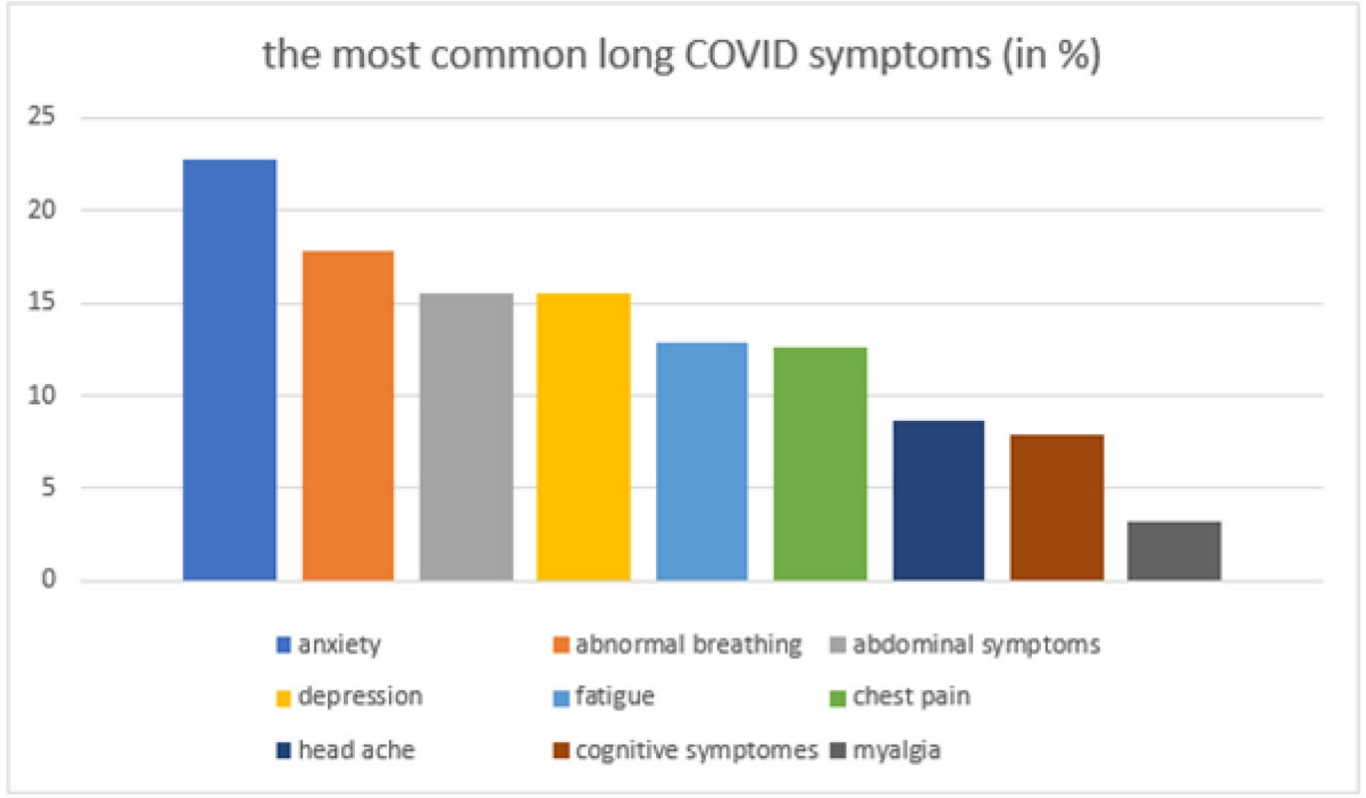
Frequency of the most common long COVID symptoms according to Taquet and his associates

The most common long COVID symptoms are presented in Fig. 2.

## Conclusions

Multiple factors seem to connect long COVID and Alzheimer’s disease. Firstly, patients with Alzheimer’s disease have an increased risk of SARS-CoV-2 infections as increased levels of ACE2 are observed in that group. On the other hand, the infection itself can promote Aβ generation, which can increase the risk of AD. There are also some similarities when it comes to molecular pathways observed in both conditions, which are misregulations in folate-mediated one-carbon metabolism (FOCM), a deficit of Cq10, and disease-associated microglia. Connections might also be found in medical imaging, such as EEG, MRI, and FDG PET. These show a decrease in the volume of gray matter, global brain size reduction, and hypometabolism in the parahippocampal gyrus, thalamus, and cingulate cortex. Additionally, a similar approach to treating AD and long COVID can be seen. It includes the use of amino adamantanes and phenolic compounds of rosemary.

In conclusion, there are a lot of connections between AD and long COVID but there is a need for further study to confirm their significance and possible application in medical practice. Hopefully, they will help medics to better understand the mechanisms and propose effective treatment for long COVID which has become a worldwide medical problem after the SARS-CoV-2 pandemic.

## Data Availability

All the research used in the creating of this article are cited in the References with doi numbers which allow to find them in the Internet.
