# Correction to: Molecular cross‑talk between long COVID‑19 and Alzheimer’s disease

**DOI:** 10.1007/s11357-024-01271-4

**Published:** 2024-07-09

**Authors:** Magdalena Pszczołowska, Kamil Walczak, Weronika Miśków, Katarzyna Antosz, Joanna Batko, Julia Karska, Jerzy Leszek

**Affiliations:** 1 Faculty of Medicine, Wrocław Medical University, Wrocław, Poland; 2 Clinic of Psychiatry, Department of Psychiatry, Medical Department, Wrocław Medical University, Wrocław, Poland

**Correction to: GeroScience (2024) 46:2885–2899**

10.1007/s11357-024-01096-1

In the original version of this article, the family name of the third author were incorrectly captured.

The third authorname should be corrected from “Weronika Misków” to “Weronika Miśków”.

The corrected authorname is shown in the authorgroup.

